# Effect of Abdominoplasty in the Lipid Profile of Patients with Dyslipidemia

**DOI:** 10.1155/2013/861348

**Published:** 2013-05-28

**Authors:** Guillermo Ramos-Gallardo, Ana Pérez Verdin, Miguel Fuentes, Sergio Godínez Gutiérrez, Ana Rosa Ambriz-Plascencia, Ignacio González-García, Sonia Mericia Gómez-Fonseca, Rosalio Madrigal, Luis Iván González-Reynoso, Sandra Figueroa, Xavier Toscano Igartua, Déctor Francisco Jiménez Gutierrez

**Affiliations:** Plastic Surgery Department, Hospital Civil de Guadalajara Fray Antonio Alcalde, Calle Hospital 278, 44280 Guadalajara, JAL, Mexico

## Abstract

*Introduction*. Dyslipidemia like other chronic degenerative diseases is pandemic in Latin America and around the world. A lot of patients asking for body contouring surgery can be sick without knowing it. *Objective*. Observe the lipid profile of patients with dyslipidemia, before and three months after an abdominoplasty. *Methods*. Patients candidate to an abdominoplasty without morbid obesity were followed before and three months after the surgery. We compared the lipid profile, glucose, insulin, and HOMA (cardiovascular risk marker) before and three months after the surgery. We used Student's *t* test to compare the results. A *P* value less than 0.05 was considered as significant. *Results*. Twenty-six patients were observed before and after the surgery. At the third month, we found only statistical differences in LDL and triglyceride values (*P* 0.04 and *P* 0.03). The rest of metabolic values did not reach statistical significance. *Conclusion*. In this group of patients with dyslipidemia, at the third month, only LDL and triglyceride values reached statistical significances. There is no significant change in glucose, insulin, HOMA, cholesterol, VLDL, or HDL.

## 1. Introduction

Dyslipidemia is a silent pandemic affecting millions of people around the world. There is more than one factor predisposing this serious problem, where not only diet, exercise, and medications could solve it [1]. 

The truth is that a lot of people can be sick without knowing it. There is controversy of the possible benefit of liposuction or abdominoplasty in the metabolism of glucose or cholesterol. There are no reports about the effect of abdominoplasty in the metabolism of patients with dyslipidemia. 

## 2. Objectives

Observe any possible change in the lipid profile, weight, cardiovascular risk markers (HOMA), glucose, or insulin of patients with dyslipidemia after an abdominoplasty. 

## 3. Methods

A descriptive observational study was designed to follow up the lipid profile of patients with dyslipidemia candidates to a body contouring surgery as abdominoplasty. The research project was evaluated and approved by the ethics and research committee of the Antiguo Hospital Civil de Guadalajara (file number in the institution 112-11). The ethics and research committee evaluated all the research projects in the decentralized, academic, and public Antiguo Hospital Civil de Guadalajara. It follows the guidelines according to the Health Mexican Norm and the Helsinki ethical principles. 

Abdominoplasty or lipoabdominoplasty is offered to women to improve the body images in case of severe skin laxity, excess fat, and flaccidity of the abdominal muscle [2, 3]. We did not operate patients with morbid obesity, where gastric bypass and other bariatric surgeries are suggested.

The following criteria for recruitment were applied patients with recent diagnoses of dyslipidemia with severe laxity of the skin, fat and musculofascial system in the lower and upper abdomen. We excluded patients with negative to participate, alterations in the morphology of abdominal wall (multiples surgical scars and defect on the abdominal wall), pregnancy, systemic illnesses that can put in risk the life of the patient (hepatic, renal or hearth problems), anomalies in the coagulation profile as antiphospholipid syndrome, procoagulants, prothrombotic disorders, primary dyslipidemia, age less than 20 years or more than 60 years and patients that had a previous body countouring surgery as liposuction, fat injection or abdominoplasty. 

 Demographic variables as age and gender were reported, as well as the fat tissue weight removed after the abdominoplasty. We calculated the sample size taking in account the number of patients operated for an abdominoplasty in one year in the institution (academic, tertiary hospital) with the help of the program in the web page http://www.macorr.com/sample-size-calculator.htm. The sample size was calculated in 17 patients. We collected patients during one year period from October 2010 to September 2011.

We observed before and three months after the surgery any possible change on weight, body mass index, laboratories values as total cholesterol, HDL, LDL, VLDL, triglycerides, hemoglobin, hematocrit, leukocytes, platelet, glucose, urea, creatinine, insulin, albumin, TGO, TGP, and HOMA index (insulin × glucose)/22.5. HOMA index is a cardiovascular risk marker. We used the Student's *t* test to evaluate any possible change before and three months after the surgery. We considered a *P* value less or equal to 0.05 as statistical significant. 

The patients were followed before and during the postoperative period in junction with the Department of Endocrinology of the same hospital. It was suggested not make any change on diet, exercise, or medications to lower cholesterol or any element in the lipid profile. A questionnaire was applied before and at the third month to evaluate the calorie intake. In the questionnaire, the last three days of food intake were evaluated. The patients were asked about their daily activities in order to identify any possible change in exercise that can increase the calories used. Three months after the surgery, patients continued followup of the dyslipidemia in the Endocrinology Department. They were advised to continue regular consults about cholesterol disorder. Medications and changes in life style as diet and exercise were started. 

## 4. Results

We operated 26 female patients between 26 and 56 years old. The mean age was 39 years old. The mean length was 1.6 meters (1.46–1.75 meters SD 0.32), weight of 69.1 kgs (54–83 kgs, SD 8.09), and body mass index of 27.4 kgs (22–30.8 kgs, SD 1.1). 

Before the surgery, the mean glucose value was of 91.45 mg/dL (72–114 mg/dL, SD 9.99), insulin value of 17.11 UI/mL (2–96 UI/mL, SD 23.38), and the HOMA index was 3.96 (0.41–24.33, SD 5.43). 

The mean hemoglobin value was 13.99 g/L (11.82–16.3 g/L SD 1.22), hematocrit 42.13 (37.2–47 SD 2.86), leukocytes count 7.33 (4.33–10.7, SD 1.87), and platelets 316 (220–440 SD 56.7).

The mean creatinine value was 0.67 mg/dL (.3–.94 mg/dL, SD 0.54), urea 20.34 mg/dL (10.7–33.2 mg/dL SD 2.2), albumin 4.11 mg/dL (3.9–6.9 mg/dL SD 0.62), DHL 175 (109–283 SD 43.72), TGO 27 mg/dL (16–45 mg/dL SD 6.6), and TGP 28 mg/dL (11–43 mg/dL SD 7.65). 

**Figure 1:**
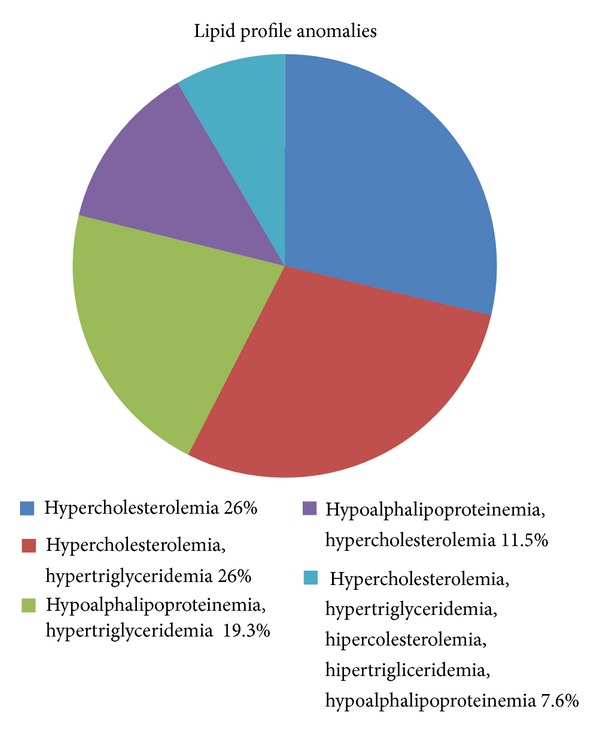


Of the 26 patients, we found 16 of the patients with more than one anomaly in the lipid profile. Sixteen of them had hypercholesterolemia, twelve had hypertriglyceridemia, nine hypoalphalipoproteinemia, and four had hiperprebeta. The results are shown in Figure 1. 

The medical treatment, diet and exercise were started by the endocrinologist at the third month of the surgery. Questionnaires were applied in order to evaluate any possible change on diet during the three-month period of time. The patients reported no change on diet. Results are shown in Table 1. 

The fat tissue removed weight between 500 and 4000 gr (mean 1700 gr).

The results before and after the surgery in weight, body mass index, total cholesterol, HDL, LDL, VLDL, triglycerides, hemoglobin, hematocrit, leukocytes, platelet, glucose, urea, creatinine, insulin, albumin, TGO, TGP, and HOMA index are shown in Table 2.

## 5. Discussion

The resection of fat tissue has consequences in the metabolism of patients. It is proved that abdominoplasty improves the metabolism of glucose, lipids, and fatty acid. Andreas and cols showed in this report that body mass index, waist/hip radio, fat mass, fat free mass, fasting plasma glucose, 2-h plasma glucose, triglycerides, total cholesterol, free fatty acids, and systolic and diastolic blood pressure decreased after abdominoplasty [4]. Something important to mention is that the reduction in the values is noticeable and the period of evaluation was longer than one month (40 days). Most of the patients were healthy with no previous impairment in weight, glucose, or any other chronic degenerative diseases. The variables before and after the surgery reached statistical significance and were between normal ranges. The same group evaluated the effect of liposuction in the metabolism of the patients [5]. It is a longer lasting report (followup at the 21 day and 90 day) that showed change in body mass index, waist hip radio, body fat, plasma insulin, triglycerides, total cholesterol, free fatty acid, systolic and diastolic pressure, inflammatory markers, leptin, TNF alfa, adiponectines, resistin, IL6 and IL10 levels. The explanation by the authors is the reduction of fat and consequently the reservoir of cytokines that improved the metabolism of the adiponectines, by decreasing the number of receptors in the fat [5, 6]. When the same group was evaluated in a randomized study, the effect of liposuction and no liposuction positive effect in insulin resistance and circulating markers of vascular inflammation were observed [6]. Our report includes patients with a previous anomaly in the lipid profile, and we excluded any patient with possible disease that affects glucose, insulin, or any other chronic illness that can bias our results; for example, in the case of medications to control glucose as metformin it can improve the lipid profile and changed our results. As another authors suggested, we had the hypothesis that in patients with lipid profile anomaly the abdominoplasty or liposuction can reduce the metabolism of cholesterol. For us, the explanation can be the effect of resistin, a protein secreted by fat tissue, which increases the production of LDL in human liver cells and also degrades LDL receptors in the liver. Consequently, removing fat tissue from abdominoplasty or liposuction can affect the secretion of resistin and decreased the production of LDL by the liver. 

Cholesterol disorders are common and asymptomatic problem in our society. Atherogenesis is strongly related with these anomalies. In Mexico it is estimated that half of the population in some parts can be affected without knowing it [1]. As other authors reported it, we did not find a benefit in other variables of the metabolism of the cholesterol as total cholesterol, VLDL, or HDL. A possible explanation is that our population had disorders of metabolism of cholesterol with higher values than other reports. For this reason, significant statistical change could be more difficult to reach in all the variables. 

Most of the patients can feel motivated by a plastic surgery so unconsciousness possible changes on diet, exercise, or life style can be done and that can explaine the positive impact. We estimated the diet before and after the surgery, and we did not find any statistical change. Interestingly, we did not notice any possible statistical change on the weight of the patients. This fact supports the thinking that if we do not modify the habits and costumes of the patients, the remaining fat tissue can hypertrophy and compensated the initial benefit of the surgery. Plastic surgery changes body images of the patients but more complex mechanisms are involved in the metabolism of the cholesterol, and so if we do not modify them plastic surgery benefit can be finished. 

We have seen an improvement but the statistical significance was not reached in other variables that were not the primary concern as, for example, insulin and HOMA. A bigger sample can clarify this fact. There are some reports that denied any possible benefit but in some of them the size of the sample is not big enough to rule out any positive benefit [7, 8]. 

Much of the recent evidence showed a positive impact in these variables [9–12]. We cannot assume that only plastic surgery can improve a complex mechanism of glucose metabolism. As we mentioned before more factors are involved and they should be modified as well. Chronic degenerative diseases are part of the interaction with the environment where less exercise and less healthy food are involved as well as genetics. When we discussed with the patients the evidence of any other possible benefit besides the change in body image we should mention that plastic surgery is not going to change a healthy life style and regular followup with the primary care physician, but that a benefit in the metabolism of glucose and cholesterol has been reported. 

In our city, previous reports were done in the case of abdominoplasty, liposuction, or combination of both methods [13, 14]. Significant improvement is noticed. Cholesterol and triglycerides are related with epiandroteniona, and the reduction of the peripheral fat decreased the levels of leptine and as consequence the levels of glucose, insulin, and cholesterol improved. Our observation is unique in evaluating Mexican patients with a documented anomaly in the lipid profile. The followup was longer almost 90 days and the results showed clinical changes with only statistical change on LDL and triglycerides. But not all the reports showed a possible benefit. 

Something important to remember is that the most dangerous fat tissue is located inside the body where body contouring surgery does not have any effect [8, 15].

This surgery is not a good choice in morbid obesity patients. But in the case of massive weight lost after a bariatric procedure, the resection of the redundant tissue, besides the functional benefit, can have an extra benefit in reducing inflammatory markers [16]. At the end, we would like to comment on the report of Swanson, one of the biggest reports about this matter. He found a positive effect in liposuction and abdominoplasty in the lipid profile of his patients. He included 322 patients. He found a difference in triglycerides values and leucocytes count. He did not find any benefits in cholesterol, VLDL, LDL, and HDL. The group of patient was a combination of different procedures (no homogeneous about the type of surgery) but the size of the group is one of the biggest reports in the literature [17].

## 6. Conclusion 

We found a reduction in triglycerides and LDL. We did not find any positive benefit in cholesterol, HDL, and VLDL, as well as hemoglobin, hematocrit, leukocytes, glucose, insulin, HOMA index, TGO, TGP, albumin, body mass index, or weight. 

Most of the patients in this group have more than one anomaly in the lipid profile. Hypercholesterolemia, hypertriglyceridemia, and hypoalphalipoproteinemia are the most common anomalies, different from reports in Mexican population. Chronic degenerative diseases are pandemic problem, where genetics is not only involved. 

Beside the possible motivation after body contouring surgery, the fat tissue that the plastic surgeons remove can have an impact on the cholesterol metabolism (especially in triglycerides and LDL). But we should be careful with this finding. If diet, life style, and exercise are not modified, the remaining fat tissue could increase in size and any positive effect can be finished.

## Figures and Tables

**Table 1:** Caloric intake estimated.

	Kcal before the surgery	Kcal three months after the surgery	*P* < 0.05
Carbohydrates	893	921	0.67
Lipids	713	732	0.42
Proteins	649	658	0.78

Total	2234	2311	0.85

**Table 2:** It shows media, range, and standard deviation before and three months after the surgery.

Variable	Before the surgery media, range and SD	Three months after the surgery media, range, and SD	*P* value, standard deviation
Weight	69.1 kgs (54–83 kgs, 8.09)	68.62 kgs (54–83 kgs, 8.07)	0.79 (8.01)
Body mass index	27.4 (22–30.8, 1.1)	27.1 (24.4–28.7, 1.32)	0.81 (1.3)
Glucose	91.45 mg/dL ( 72–114 mg/dL, 9.99)	90.71 mg/dL (76–106 mg/dL, 8.77)	0.27 (9.32)
Insulin	17.11 UI/mL (2–96 UI/mL, 23.38)	11.79 UI/mL (3–57.4 UI/mL, 11.15)	0.28 (18.3)
HOMA	3.96 (0.41–24.33, 5.43)	2.58 (0.7–10.67, 2.3)	0.22 (4.1)
Hemoglobin	13.99 mg/dL (11.82–16.3 mg/dL, 1.22)	12.79 mg/dL (11–15.3 mg/dL, 1.06)	0.1 (1.2)
Hematocrit	42.1 (37.2–47, 2.86)	42.13 (37–43, 2.8)	0.3 (2.8)
DHL	175 (109–283, 43.72)	178 (110–296, 52.15)	0.83 (47)
TGO	27 mg/dL (16–45 mg/dL, 6.6)	28.32 mg/dL (12–43 mg/dL, 8.05)	0.74 (7.5)
TGP	28 mg/dL (11–43 mg/dL, 7.65)	31.79 mg/dL (14–49 mg/dL, 7.89)	0.33 (8)
Albumin	4.11 mg/dL (3.9–6.9 mg/dL, 0.62)	3.8 mg/dL (2.8–5.3 mg/dL, 0.64)	0.094 (0.64)
Cholesterol	224 mg/dL (134–488 mg/dL, 69.55)	220 mg/dL (128–446 mg/dL, 62.56)	0.84 (65)
Triglycerides	193 mg/dL (61–369 mg/dL, 51.2)	133 mg/dL (26–286 mg/dL, 80.75)	0.03 (73.2)
HDL	44 mg/dL (6–69 mg/dL, 10.99)	49 mg/dL (32–38.6 mg/dL, 29.6)	0.18 (23.2)
VLDL	43 mg/dL (12–133 mg/dL, 26.1)	39.1 mg/dL (11.8–122 mg/dL, 22.04)	0.55 (23.85)
LDL	137 mg/dL (130–390 mg/dL, 68.43)	97.61 mg/dL (26–295 mg/dL, 71.86)	0.04 (72.33)
